# Nonlinear time-series approaches in characterizing mood stability and mood instability in bipolar disorder

**DOI:** 10.1098/rspb.2011.1246

**Published:** 2011-08-17

**Authors:** M. B. Bonsall, S. M. A. Wallace-Hadrill, J. R. Geddes, G. M. Goodwin, E. A. Holmes

**Affiliations:** 1 Mathematical Ecology Research Group, Department of Zoology, University of Oxford, Oxford OX1 3PS, UK; 2 St Peter's College, New Inn Hall Street, Oxford OX1 2DL, UK; 3 Department of Psychiatry, Warneford Hospital, University of Oxford, Oxford OX3 7JX, UK

**Keywords:** nonlinear time-series analysis, autoregressive approaches, mood variability, cognitive neuroscience, bipolar disorder

## Abstract

Bipolar disorder is a psychiatric condition characterized by episodes of elevated mood interspersed with episodes of depression. While treatment developments and understanding the disruptive nature of this illness have focused on these episodes, it is also evident that some patients may have chronic week-to-week mood instability. This is also a major morbidity. The longitudinal pattern of this mood instability is poorly understood as it has, until recently, been difficult to quantify. We propose that understanding this mood variability is critical for the development of cognitive neuroscience-based treatments. In this study, we develop a time-series approach to capture mood variability in two groups of patients with bipolar disorder who appear on the basis of clinical judgement to show relatively stable or unstable illness courses. Using weekly mood scores based on a self-rated scale (quick inventory of depressive symptomatology—self-rated; QIDS-SR) from 23 patients over a 220-week period, we show that the observed mood variability is nonlinear and that the stable and unstable patient groups are described by different nonlinear time-series processes. We emphasize the necessity in combining both appropriate measures of the underlying deterministic processes (the QIDS-SR score) and noise (uncharacterized temporal variation) in understanding dynamical patterns of mood variability associated with bipolar disorder.

## 1. Introduction

Bipolar disorder is a mental disorder characterized by manic episodes of elevated mood and overactivity, interspersed with periods of depression [1]. Approximately 1 per cent of adults in the general population have a lifetime prevalence of bipolar disorder [2]. It is a major cause of morbidity and mortality owing to suicide [3], with the highest rate of suicide of all the psychiatric disorders [4]. Bipolar disorder is highly recurrent and typically a chronic, lifelong condition. In the past, recovery from individual manic and depressive episodes was seen as a hallmark feature which classically distinguished even severe bipolar illness from schizophrenia. This perspective has shifted with the recognition that ongoing mood variation may lead to chronic functional impairment. Mood stability, rather than episodes of mania or depression, has assumed increasing importance in our understanding of the disorder [5,6] and its development during childhood [7]. Nevertheless, until recently it remained difficult to measure mood stability and so, despite its apparent importance, we know strikingly little about the nature of bipolar mood variation over time in patients.

The need to understand the nature of mood variation over time in bipolar disorder is made more urgent by the translational need to innovate better treatments based on insights from cognitive neuroscience. Hitherto, pharmacological treatments have been the primary approach for bipolar disorder based on their ability to shorten acute episodes and in the long term to prevent relapse [8]. For example, treatment with lithium, established over 40 years ago, remains the benchmark mood stabilizer, but has moderate efficacy, being fully satisfactory in a minority of patients [9], and reducing relapse rates by 30–40% [10]. Developments in pharmacological treatments have substantially increased the number of options but remain moderate in efficacy [11]. A substantial proportion of patients remain both symptomatic during inter-episode periods, and at a high risk of relapse [5].

Psychological treatments have been developed as adjunctive treatments for the management of bipolar disorder. Those tested in randomized control trials include: family-focused therapy [12,13], interpersonal social rhythm therapy [14,15], psychoeducation [16] and cognitive behaviour therapy [17,18]. However, even when combining pharmacological and psychological interventions, ongoing inter-episode dysfunction and relapse rates remain high [19,20].

**Figure 1.**
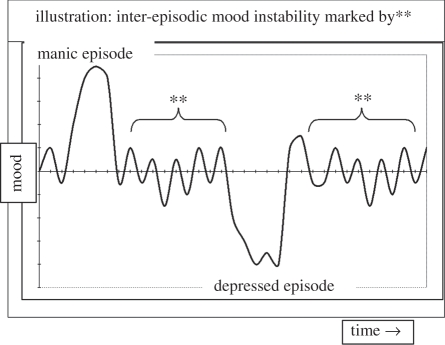
A schematic of mood patterns in bipolar disorder: the disorder does not simply feature full-blown episodes of mania and depression with periods of normality. Rather, ongoing inter-episodic mood instability is also a clinically common, yet a poorly understood feature of the disorder.

We have proposed that treatment innovation drawing on new insights from cognitive neuroscience could move the field to a new level (e.g. [21]). We are struck that almost all bipolar treatment research have been exclusively episode-focused, so directed either to shortening or preventing the recurrence of full-blown episodes of mania/depression. This imposes inherent and major limits on what research is feasible because episodes are relatively infrequent; in the case of full-blown manic episodes, the median hospital re-admission time after a first episode is 4 years [22,23]. Clinical trials are accordingly lengthy and expensive and proof-of-concept of a new treatment is difficult to establish by any other means. The solution may be to address the emotional dysfunction on a continuum, to allow consideration of ongoing mood variability as an alternative and clinically significant target [6]. Many patients experience significant day-to-day or week-to-week mood swings below the criteria for a full-blown episode, but above those experienced by non-affected individuals. This mood instability over time impairs daily functioning, yet remains understudied [24]. See figure 1 for a schematic of bipolar mood instability. The development of symptom-specific treatments [25,26], for example, to preserve inter-episodic mood stability [5,27] would promise a major effect on the quality of life.

We have been monitoring mood symptoms on a weekly basis in patients with bipolar disorder in our clinic for several years. This is done by the use of a locally developed computerized mobile phone short message service (SMS; text messaging) by which patients complete weekly measures of depressed and manic mood [28–30]. Clinically, the interpretation of data is simply done by visual inspection by practicing clinicians. However, given the temporal nature of this sort of data, here we develop specific hypotheses based on these time-series fluctuations with the aim of statistically understanding mood variability. Several studies suggest that many psychiatric conditions, including bipolar disorder are nonlinear over time [31,32]. Thus, change in mood cannot simply be predicted from simple linear relationships and developing methods for the analysis of longitudinal patient data will complement information from traditional statistical analyses [33]. Nonlinear time-series analysis is a well-developed field of statistical research (e.g. [34]), and we extend these methods (see the electronic supplementary material) to explore mood variability in patients diagnosed with bipolar disorder.

**Figure 2.**
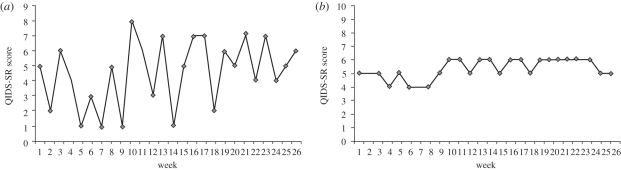
Illustration of the type of mood score charts used to rate patients in clinic as either stable or unstable. Mood stability in bipolar disorder was assessed over the course of six months using depressed mood score data (QIDS-SR) for individuals and used to define as either (*a*) an ‘unstable’ mood profile or (*b*) a ‘stable’ mood profile.

In an initial study, we tested psychological differences between our patients clinically classified as having an unstable as opposed to stable mood profile [35]. Twenty-three patients with bipolar disorder each provided weekly mood ratings through mobile phone SMS as a part of their ongoing clinical care. For each patient, these data yielded a pattern of mood fluctuation over time (figure 2). These charts, showing approximately six months of data, were independently categorized via clinical visual inspection by two psychiatrists for mood instability, yielding two groups: stable (*n* = 11) versus unstable mood (*n* = 12). While we have corroborated these clinical decisions of mood stability using simple statistical analyses [35], a key question that emerged was whether this clinically compelling division of mood stability could be understood more thoroughly using a longitudinal or time-series approach.

Thus, the main aim of the present study was to use a nonlinear time-series analysis approach to explore the variability in weekly mood scores of patients with bipolar disorder collected over a long time period of up to 4 years and 12 weeks. Since in our clinical practice patients spend more time in depressed rather than in manic mood states [28,36,37], and that depressed mood is used as the key barometer in patients' care, we focused on depressed rather than on manic mood scores. The scores of patients divided into mood stable and unstable groups by clinical judgement were examined to explore this classification in characterizing the variability in patient mood within and between groups. Linear and nonlinear time-series models fitted to the two patient groups reveal distinct differences in the temporal patterns associated with mood. These results are discussed in the light of recent developments in the role of cognitive behavioural therapies in bipolar disorder.

## 2. Material and methods

### (a) Participants

Twenty-three patients (10 females and 13 males; mean age = 44.4, s.d. = 11.8) with bipolar disorder (Diagnostic and Statistical Manual of Mental Disorders, 4th edn, Text Revision (DSM-IV-TR); [1]) from a local mood disorders clinic were recruited for this study [35]; for further demographic and clinical details see table 1*a*. It was established that at the time of baseline participation and by clinician judgement (see below), all individuals were either euthymic or experiencing low levels of mood fluctuation (i.e. not in a distinct episode or in need of in-patient hospital care). Participants also reported no current alcohol or drug misuse and no diagnosis of schizoaffective disorder. All participants gave written informed consent and completed standardized paper questionnaires for baseline assessment; these measured levels of depression (QIDS-SR; [38]), mania (Altman self-rating scale for mania (ASRM); [39]) and trait anxiety (Spielberger state anxiety inventory (STAI); [40]), as detailed in table 1*a*.

**Table 1.** (*a*) Demographic and clinical details: means and standard deviations (in brackets) for the bipolar stable and unstable groups for age, gender and baseline measures of depression (QIDS-SR, quick inventory of depressive symptomatology-self-rated; [38]), mania (ASRM, Altman self-rating scale for mania; [39]) and anxiety (STAI, Spielberger state anxiety inventory; [40]). (*b*) QIDS-SR depressed mood scores over the full time series: descriptive statistics for each group separately including means and s.d. of stable and unstable groups and total number of SMS replies containing the QIDS-SR data sent by the patients (see also electronic supplementary materials, table A2).

	bipolar stable mood (*n* = 11)	bipolar unstable mood (*n* = 12)
(*a*) *demographic and clinical details*		
age in years (s.d.)	41.64 (12.74)	47.00 (10.80)
gender (female : male)	5 : 6	5 : 7
baseline depression (s.d.)	4.17 (3.66)	12.83 (6.70)
baseline mania (s.d.)	3.45 (3.42)	2.17 (2.17)
baseline anxiety (s.d.)	33.45 (6.93)	51.08 (12.70)
bipolar subtype		
bipolar I	9	12
bipolar II	2	0
(*b*) *QIDS-SR depressed mood scores over the full time series*		
mean	4.06	11.01
s.d.	3.41	6.48
median	3	10
skewness	1.12	0.58
kurtosis	4.00	2.43
total number of SMS	1410	1791

### (b) Division of patients into stable versus unstable mood groups

Patients were divided into two clinically relevant groups based on an initial baseline period of (approx.) six months of mood score data (see below) from which their mood stability was assessed. Patients were categorized into one of two groups, stable (five females and six males) or unstable (five females and seven males) using both this clinical judgement of mood score charts (figure 2) and non-parametric statistical analyses (see [35]). Participant characteristics are detailed in table 1(*a*) with further details concerning bipolar disorder history in electronic supplementary material, table A2.

### (c) Method of mood score data collection using mobile phone technology in daily life

The QIDS-SR [38] consists of a 16 item questionnaire measuring severity of depression, covering the nine DSM-IV-TR [1] major depressive disorder symptoms. Patients were asked to choose the response that best described themselves over the past 7 days on a four-point scale (0–3) anchored at all points by a description. For example, Question 5, ‘feeling sad’ is anchored at 0 = ‘I do not feel sad’, 1 = ‘I feel sad less than half the time’, 2 = ‘I feel sad more than half the time’ and 3 = ‘I feel sad nearly all the time’. While scores on the QIDS-SR can be also clinically grouped into five severity levels: none (0–5), mild (6–10), moderate (11–15), severe (16–20) and very severe (21–27), here we consider that scores vary continuously across this scale (0–27). The QIDS-SR has established psychometric properties for rating depressive symptom severity in individuals with bipolar depression [41] as well as chronic major depressive disorder [38]. It is known to be strongly positively correlated with clinician-reported scales [42,43].

Weekly mood score data on the QIDS-SR were collected through the SMS system developed in the local Mood Disorders clinic [28,29] to generate the aforementioned clinically useful charts. Patients were given credit card-sized versions of the QIDS-SR scale. A bespoke computer program automatically sent out weekly text messages to patients to prompt them to submit their self-rating. To do this, patients simply replied with a text message containing a list of numbers corresponding to their self-rating on each of the QIDS-SR items.

The present study used a real-world clinical setting in which participants began using the SMS system at different points and for differing lengths of time. The QIDS-SR data for the time-series analysis were collected between 21 December 2006 and 7 March 2011. The minimum time any patient contributed texts was 46 weeks and the maximum time 220 weeks.

Infrequently, patients replied with more than one text per week. The first valid response to the weekly prompt was used in the analysis, and subsequent responses within the week removed. If no valid response was received within the week following the prompt, this was coded as missing data up until a patient's last actual response to the system. There was a total of 648 non-responses (stable *n* = 455 and unstable *n* = 193). In addition, the SMS system occasionally did not send prompts (e.g. if a participant requested temporarily to take a break from texting). Weeks in which no prompt was sent were also coded as missing data (total missing, *n* = 101; stable, *n* = 74; unstable, *n* = 31).

### (d) Data analysis

#### (i) Descriptive statistics

We analysed the full dataset by group (stable/unstable) to establish the mean and standard deviations of the QIDS-SR weekly mood score, the median score and the skew and kurtosis of the scores. Levene's test of homogeneity of variance was used to establish equivalence of variance between the two groups. All analyses were undertaken in *R* [41].

#### (ii) Missing values analysis

To describe the patterns in the missing values and develop appropriate approaches for the time-series analysis (see below) the following analyses were used. The attrition rate of patients from each group was analysed using survival analysis to establish whether individuals were more likely to ‘drop-out’ (i.e. stop sending in weekly mood scores) in one group compared with the other.

Using the QIDS-SR score or missing value, we conducted binary regressions to determine if the distribution of missing values was dependent on individual patients or group (stable/unstable). Analysis was conducted using all the QIDS-SR data (220 points per patient). This was regressed against group or patient number. Further analyses were undertaken to see if the patterns in missing values depended on time; in particular, analyses based on the time interval between missing values (waiting time until the next missing value) were undertaken using individual patients or mood group classification in a generalized linear modelling approach. Again, all analyses were undertaken in *R* [41].

#### (iii) Time-series analysis

Based on a conditional likelihood framework [44], we fitted linear and nonlinear (threshold based) autoregressive (AR) models to the QIDS-SR score data for each patient within each clinically allocated group (details are given in the electronic supplementary material). The baseline AR model (AR(1)) takes the form:2.1
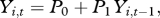
where *Y*_*i,t*_ is the QIDS-SR score at time *t* for the *i*th patient, *P*_0_ and *P*_1_ are model parameters (that are to be estimated using the likelihood-based framework). Given the non-normal distribution of mood scores (see electronic supplementary material, figure A1), we assumed that the errors associated with our AR processes (e.g. equation (2.1)) were gamma-distributed (see the electronic supplementary material).

Using the last actual response as the end of any given patient's time series, we fitted a range of different AR models to the (replicated) patient mood scores, including linear AR(1) and AR(2), threshold AR(1) and threshold AR(2) (where the threshold is based on the mean score for each patient) and group-structured AR models (where the potential for different AR time-series processes between the two different groups is tested). To evaluate goodness of fit, we used the Akaike information criterion (AIC; [45]) to discriminate weights of evidence for competing hypotheses [46] and hence the different time-series model structures on mood variability. For comparisons of individual patient trajectories to the best fitting model, we computed root mean-squared error (RMSE) values as a measure of goodness of fit. Analyses were conducted in C (see the electronic supplementary material; code available on request).

## 3. Results

### (a) Descriptive statistics

Demographic and clinical details of the patients (including age, baseline mood scores, and further details concerning bipolar disorder history) are described in table 1*a*. Table 1*b* shows the QIDS-SR-depressed mood scores over the full time series. As shown in table 1*b*, and as might be anticipated, mean (and median) QIDS-SR mood scores were higher and scores demonstrated more variability in the unstable group of patients compared with the stable group (see also figure 2 for an illustration). Levene's test confirmed that there was a significant difference in the variances between the two groups (*F* = 571.93 on 1 and 3199 d.f.; *p* < 0.001). The distribution of scores (see electronic supplementary material, figure A1) was non-normal with positive skew and leptokurtic levels of kurtosis.

### (b) Missing values analysis

**Figure 3.**
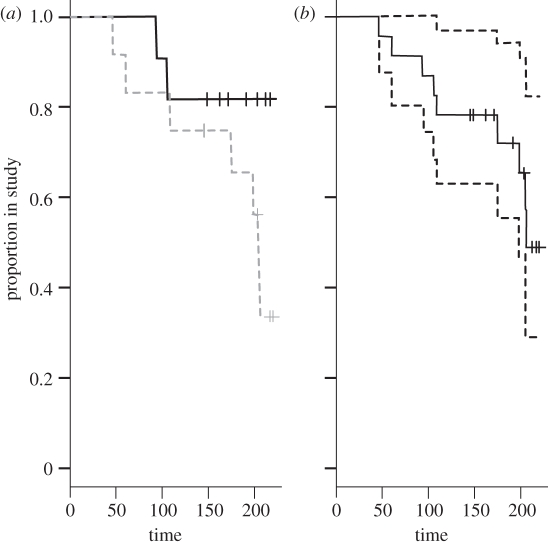
Patient attrition rates from the study over 220 weeks. Plot showing (*a*) survival curves for the stable (solid lines) and unstable (dashed lines) group and (*b*) the overall survival group (with confidence intervals as dotted lines).

Although fewer patients left from the stable group (greater than 80% remained in the study after 220 weeks) than the unstable group (less than 30% of patients remained in the study after 220 weeks), there was no significant difference in patient attrition rates (figure 3) between the two groups (*χ*^2^ = 1.9 on 1 d.f.; *p* = 0.17). This suggests that there is no evidence of systematic differences in the distribution of missing values between the two groups of patient.

While both patient and grouping classification were predictors of missing values, the effect of patient was the strongest (largest reduction in deviance—*χ*^2^ = 1805.2 on 22 d.f.; *p* < 0.001) compared with group (*χ*^2^ = 49.84 on 1 d.f.; *p* < 0.001)—missing values varied idiosyncratically across patients. Generalized mixed models demonstrated that the missing values were random through time but varied among patients. Waiting times were distributed exponentially with mean time between missing values of 4.83 weeks (±1.102 weeks; 95% CI). Waiting times between missing values were also more appropriately accounted for by including covariates; including patient rather than group was a better descriptor of the distribution of waiting time between missing values (AIC_group_ = 3289.855 versus AIC_patient_ = 3157.156).

### (c) Time-series analysis

As missing values were randomly distributed between groups and over time, we used an expectation–maximization algorithm to fit the range of time-series models (see the electronic supplementary material) to the mood score data. Based on AIC scores (electronic supplementary material, table A3), the best fitting model included both group structure and threshold effects in the time series. Differences in AIC values (*Δ*AIC) showed that there was no support for any other model (electronic supplementary material, table A3). For the stable group, time-series patterns associated with this group of patients were best described by a threshold AR(1) process, where above the threshold (the individual patient's mean mood score) the time series are predicted to follow:3.1
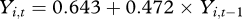
and below the individual patient's mean score, the time series follow:3.2
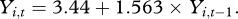


**Figure 4.**
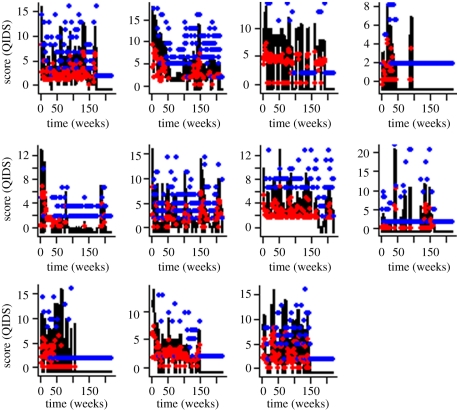
Mood score time series for individual patients from the stable group (black lines) with fitted threshold autoregressive model (equations (3.1) and (3.2); red and blue points). RMSE values (see the electronic supplementary material) are used to describe the overall correspondence between the points predicted by the equation (circles) and the actual values reported by patients (black lines). Note that the scale on the *y*-axes vary between plots.

The goodness of fit of this threshold model to patients in the stable group is illustrated in figure 4. In contrast, the time-series patterns associated with the unstable group are best described by a threshold AR(2) process, where above the mean mood score, the time series are described by3.3
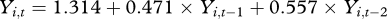
and below the mean score, time series in the unstable group follow:3.4
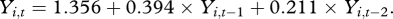

**Figure 5.**
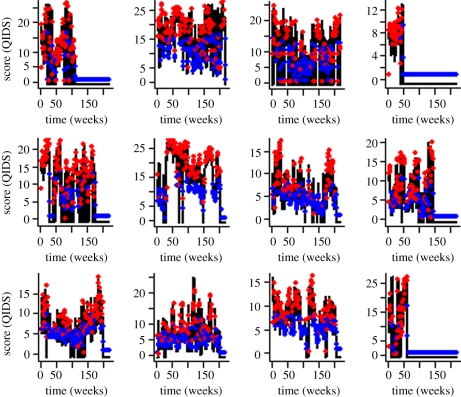
Mood score time series for individual patients from the unstable group (black lines) with fitted threshold autoregressive model (equations (3.3) and (3.4); red and blue points). RMSE values (see the electronic supplementary material) are used to describe the overall correspondence between the points predicted by the equation (circles) and the actual values reported by patients (black lines). Note that the scale on the *y*-axes vary between plots.

The goodness of fit of this threshold AR(2) model to the individual patient mood scores in the unstable group is illustrated in figure 5. RMSE values of the fit of the model to the individual patient time series showed that there was higher concordance between model and data in the unstable group (described by a threshold AR(2) model) than the stable group of patients (see electronic supplementary material, figure A2). Poorer overall lack of fit of the threshold AR(1) model to individual patients in this group contributed to the distribution of RMSE values. However, the variability in RMSE values between the two groups was higher in the unstable group of patients suggesting that long runs of missing values in some individuals within this group of patients account for this lack of fit.

## 4. Discussion

Here, we have developed a nonlinear time-series approach and shown that we can characterize mood variability in two groups of patients with bipolar disorder using particular types of time-series models based on AR processes. We show that the dynamics of mood is different between the two groups of patients; in the stable group mood dynamics are best predicted using a simple AR(1) in which only previous week mood scores influences the current score. By contrast, the mood variability in the unstable group of patients is predicted using a more complicated AR(2) model in which two weeks' previous mood scores are necessary to predict current mood score. Within the different patient groups, these AR processes differ above and below a threshold (the individual patient's mean mood score) and suggest that the observed weekly time series in both groups are highly nonlinear. The implications of these sorts of thresholds, where the (predicted) patterns of mood are different above and below a patient's mean mood score, could be used to help in classifying patients based on their mood stability and accordingly help in understanding the predicted course of their illness (e.g. AR(1) versus AR(2)).

The existence of nonlinear patterns in mood variability over time suggests a range of deterministic dynamical patterns structuring these sorts of longitudinal data. Nonlinear dynamics can range from regular periodic cyclic patterns, such as those associated with circadian rhythms through to highly aperiodic and chaotic dynamics (such as those associated with physiological processes) [47]. Some approaches to explore the potential for chaotic patterns (extreme sensitivity to initial conditions) in mood dynamics have been developed [31,32,48,49]. Patients diagnosed with bipolar disorder compared with normal subjects are more likely to show aperiodic and low-dimensional chaotic patterns in self-rated mood assessments [31]. One expectation from this is that the biological rhythms and short-term circadian patterns associated with emotions and mood are disrupted in patients suffering from bipolar disorder [50,51], and this leads to aperiodic variability in mood. While this possibility might be expected to lead to highly nonlinear time-series patterns, it would require the long-term breakdown of regular cyclic patterns in mood [52,53].

Understanding these sorts of deterministic aperiodic dynamics or chaotic dynamics necessitates a careful characterization of both the initial conditions (owing to extreme sensitivity) and structure of the dynamics. Stochastic variation (owing to inherent difficulties in precisely observing a process or inherent fluctuations that are difficult to characterize) makes accurately identifying these sorts of dynamics in many systems extremely complicated [54,55]. For our study, characterizing the dynamical patterns of mood variability associated with bipolar disorder required understanding both the contributions of the deterministic process (in this case, the QIDS-SR mood score) and stochastic variation (the noise associated with the longitudinal data, such as the nature of the missing values, the structure of the observation error and the inherent and uncharacterized error associated with patient mood variability not captured by the QIDS-SR scoring system). In beginning to unravel this, as far as we are aware, our study is the first to use an appropriate statistical time-series framework (characterizing the nature of the missing values and understanding the phenomenological stochastic errors) to understanding longitudinal patterns of real clinical data in bipolar disorder.

We show that these stochastic errors are non-normally distributed, differ between the stable and unstable groups of patients and can be appropriately described in time-series models (see the electronic supplementary material). However, characterizing this sort of noise requires appropriate consideration of the underlying biological–psychological mechanisms in order to understand not only the dynamical patterns (figures 4 and 5), but also the discrepancies between the model and the individual patient longitudinal mood patterns. While our threshold AR models describe the differences between stable and unstable groups in terms of the number of weeks need to construct an appropriate time-series model (equations (3.1) and (3.2) versus equations (3.3) and (3.4)), how the individual patient longitudinal patterns are described by these statistical models is highly variable. Greater lack of fit of the time-series models (and hence more inherent unexplained error) is observed in the stable group of patients and we attribute this discrepancy to the greater number of missing values in the time-series and intrinsic difficulties in precisely predicting mood. However, intragroup variability is greater within the unstable group owing to the longer runs of missing values observed in some patients.

That there are differences between these groups, therefore, suggests that bipolar patients' mood scores over time can be used to determine whether they will occupy stable or unstable clinical courses during their illness. This shows for the first time that we can begin to characterize mood stability in bipolar disorder by looking at fine-grained analyses of weekly mood experience. It also suggests that we may be able to build predictive case profiles for individual patients. For instance, the longitudinal nature of our study allows predictions to be made on prospective mood variability and two complementary ways to do this could be to take new patient information and infer the time-series structure (e.g. TAR(1) or TAR(2)) thus assigning the new patient to a stable or unstable group (complimentary to clinical judgement) or using the predicted models (equations (3.1) and (3.2) or equations (3.3) and (3.4)) to determine goodness of fit (e.g. *r*^2^ or mean-squared error). We emphasize that this is neither a test nor a replacement for clinician decision *per se*, but a statistical approach in understanding the nature of human mood variation over time. We also note the need to replicate this approach using a larger sample. It is also possible that characterizing the different temporal dynamics of mood variation (between groups or within individuals) could provide an alternative, shorter term measure for the outcome of treatments, for example, establishing if interventions result in a change from an unstable to stable mood profile. Future research could consider whether mood fluctuations vary differentially between and within scores that may reflect clinical criteria for full-blown depressive episodes. Note this might best be done alongside clinical judgement for each patient. Further, manic episodes, given collection of manic mood data, could also be studied.

Combining a cognitive science approach to bipolar mood experience, alongside clinical insights from psychiatry and psychology, with a statistical time-series approach offers a way to describe (and monitor) mood variation. In the absence of a way to begin to describe mood variability, we lack the framework to subject bipolar mood to the type of rigorous cognitive neuroscientific analysis required to unravel the underlying biological–psychological mechanisms driving mood variability and episodes of mania or depression. Furthermore, while the use of SMS weekly mood monitoring has been a recent and well-received clinical innovation, currently interpreting such data rely on visual inspection of scores by clinicians. The current statistical approach opens up a path to aid treatment innovation. Treatment innovation in turn should be aimed at allowing patients to experience a more stable mood and life, and we need to be able to monitor treatment impact and the course of such improvements.
